# A Comparison of Sports and Exercise Medicine Training for Physicians Across Five English-Speaking Countries

**DOI:** 10.7759/cureus.93201

**Published:** 2025-09-25

**Authors:** John Fahmy, Fady Kamel, Matthew Fahmy, Andrew Henein, Yasmeen Khan

**Affiliations:** 1 General Practice, Institute of Sport, Exercise and Health, University College London, London, GBR; 2 Trauma and Orthopaedics, Peterborough City Hospital, North West Anglia NHS Foundation Trust, Peterborough, GBR; 3 Foundation Programme, Bedford Hospital, Bedfordshire Hospitals NHS Foundation Trust, Bedford, GBR; 4 Foundation Programme, Lister Hospital, East and North Hertfordshire NHS Trust, Stevenage, GBR; 5 Trauma and Orthopaedics, North West Anglia NHS Foundation Trust, Peterborough, GBR

**Keywords:** fellowship training, international comparison, medical education, medical training, postgraduate training programs, sport and exercise medicine, sports medicine, sports medicine curriculum, training pathways

## Abstract

Sports and Exercise Medicine (SEM) has rapidly evolved into a formally recognised and important medical specialty that supports population health and tackles chronic disease burden in addition to injury prevention and optimisation of athletic performance. The specialty’s establishment has adopted different timelines globally, and training pathways differ markedly across English-speaking countries, despite common clinical goals. In this review, we aim to provide a comparison between the postgraduate training pathways for physicians in SEM across five English-speaking countries highlighting the main differences, strengths and drawbacks of each pathway. This review will be able to guide future changes in the training pathway and inform aspiring trainees considering a career in SEM. Data on pathways to board certification in SEM, training program requirements, structure, duration, examinations and competition levels was collected from literature, official governing bodies’ publicly available documents and online resources.

Postgraduate training pathways in SEM vary internationally in structure, duration and content. The UK, Australia and New Zealand recognise SEM as a stand-alone specialty delivered through nationally standardised programs, providing sustained exposure across musculoskeletal, exercise and wider population health domains. The US and Canada offer SEM as a subspecialty via shorter fellowships delivering procedural focus and increased team medicine involvement but with greater variability in content and reduced emphasis on exercise medicine. Structured programs ensure curricular consistency and depth but require longer training and face high competition for posts. Fellowship models enable faster entry to independent practice and maintenance of dual specialty roles at the risk of narrowing clinical focus. Recognising the strengths and drawbacks of each pathway can inform refinement of SEM training internationally and guide aspiring SEM physicians in selecting pathways aligned with their career goals and the demands of both training and application processes.

## Introduction and background

Sports and Exercise Medicine (SEM) is a relatively young but rapidly evolving medical discipline. Sports medicine focuses on prevention, diagnosis and management of illness and injury pertaining to physical activity. Exercise medicine utilises tailored physical activity in the prevention and management of disease, in addition to improving overall health. The conceptual foundations of SEM reach as far back as Hippocrates who taught “walking is man’s best medicine” and Galen, a Roman physician to gladiators, who declared “employment is nature’s physician” [1]. However, the field began to emerge as a distinct medical specialty in the twentieth century, driven by advances in sport science, exercise physiology, public health and elite sport, prompted by the Olympic movement and rehabilitation demands associated with wartime injuries [2]. Whilst timeframes for formal recognition of SEM as a specialty differ globally, the discipline plays a vital role in addressing the burden of non-communicable disease globally, rehabilitation from injury and illness, and optimisation of physical function in chronic disease, disability and ageing [3]. Recent advances in SEM have translated into a wide range of clinical and public health interventions, including exercise prescription for chronic disease management, neuromuscular training programs for injury prevention, return-to-play protocols and public health initiatives promoting active lifestyles [4,5].

SEM interventions are not only limited to athletic performance but have shown to improve health outcomes across a wide range of chronic conditions. Exercise prescription has been shown to reduce cardiovascular disease risk by up to 35% and improve glycaemic control in type 2 diabetes [6,7]. Furthermore, structured physical activity has proven to significantly lower the incidence and severity of depression [8]. Well-implemented injury prevention programs, such as the Fédération Internationale de Football Association (FIFA) 11+, have demonstrated reductions in sports injuries by as much as 50% and graded return-to-play protocols have become the hallmark of safe concussion management [9,10]. These outcomes reflect the value of SEM in both individual patient care and at a public health level.

Whilst SEM has grown substantially as a medical discipline, reflecting its importance in population health, there remains considerable variation in how the specialty is structured across health systems. Specialty variation extends into entry routes and training pathways presenting challenges for workforce planning and international consistency. This review aims to examine and compare the postgraduate training pathways for physicians in sports and exercise medicine across five English-speaking countries with established and reputable programs: the UK, the United States of America (USA), Canada, Australia, and New Zealand. We explore key differences between these systems, highlighting their respective strengths and challenges, with the objective of guiding future training reforms and providing guidance for prospective trainees considering a career in SEM.

## Review

A comparison review of SEM training pathways was conducted amongst five English-speaking countries with well-established programs: the United Kingdom, the United States of America, Canada, Australia and New Zealand. Data was gathered from literature, publicly accessible documents from official governing bodies, and online resources. Where multiple sources described the same training pathway, the most recent and authoritative information from accrediting bodies was prioritised. The primary variables analysed included the structure of the training pathway, training program requirements, competition rates, curriculum content, program duration and examination requirements.

United Kingdom

In the United Kingdom (UK), musculoskeletal (MSK) conditions account for 21% of primary care presentations underlining the importance of the MSK burden on clinical service delivery as well as its wider impact on the workforce and economy [11]. MSK complaints are the leading cause of work absence and reduced productivity. Back pain alone accounts for 12 million lost workdays per year [12]. The magnitude of productivity loss illustrates the public health impact and the pressing need to address it effectively. SEM serves as a vital link connecting primary care, public health and specialty medicine with the comprehensive management of MSK conditions to reduce both the clinical and public heath burden.

Training Pathway

**Figure 1:**
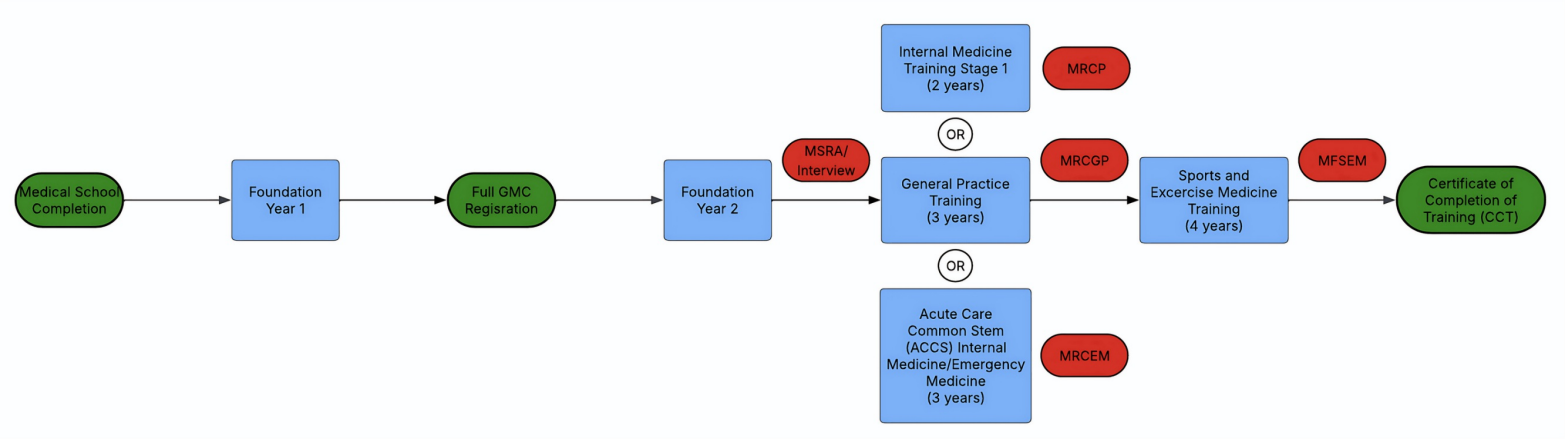
The UK Training Pathway for Higher Specialty Training (HST) in Sport and Exercise Medicine This figure illustrates the various pathways that UK medical graduates can take to enter Sports and Exercise Medicine training adapted from the Faculty of Sports and Exercise Medicine official website [13]. Following completion of the Foundation Programme, applicants will undergo the Multi-Specialty Recruitment Assessment (MSRA) and/or an interview to enter their desired training programme. They will then either complete Internal Medicine training stage 1 (2 years), General Practice training (3 years), or Acute Care Common Stem training (3 years). This involves the completion of the relevant examinations – MRCP, MRCGP and MRCEM. Following this, applicants are eligible for higher specialty training in Sports and Exercise Medicine (4 years). Upon completion of training and the MFSEM examinations, applicants will receive a Certificate of Completion of Training (CCT).

UK graduates need to undertake the common two-year foundation training pathway: Foundation Year 1 (FY1) and Foundation Year 2 (FY2). A full General Medical Council (GMC) license is obtained upon the completion of the FY1 [13]. Doctors wishing to pursue a career in Sports and Exercise Medicine need to complete one of the three core pathways to specialist training as depicted in Figure 1 by the Faculty of Sports and Exercise Medicine (FSEM) [13]. The training program is designed and delivered by FSEM and the Joint Royal Colleges of Physicians Training Board (JRCPTB).

Physician applicants must have passed the Membership of the Royal College of Physicians (MRCP) Part 1 at the time of application to be eligible and must have achieved the full diploma by the offer date. Physician applicants must also demonstrate achievement of core medical capabilities through current or prior training in recognised UK or equivalent internal medicine programmes supported by Annual Review of Competency Progression (ARCP) or an alternative certificate [14]. General practice applicants are required to have full Membership of the Royal College of General Practitioners (MRCGP) by the offer date. General practice applicants must either have obtained full GP registration or be in the final year of a GP training programme on track for completion [14]. Acute care trainees undertake the Emergency Medicine or Internal Medicine training branch of the Acute Care Common Stem (ACCS) training pathway, comprising four Specialty Trainee (ST) years. Emergency Medicine applicants must have either Membership of the Royal College of Emergency Medicine (MRCEM) or have completed the Fellowship of the Royal College of Emergency Medicine (FRCEM) Intermediate Certificate by the offer date. Emergency Medicine applicants must show evidence of completion of ST1-ST3 ACCS Emergency Medicine competencies via workplace-based assessments and ARCP or equivalent [14].

The diverse entry routes into SEM training are a major strength of the programme, reflecting the need for a broad skill set spanning primary care, secondary care and population health. Trainees currently enrolled in the specialist training program come from a diverse range of specialties, with national recruitment data indicating that roughly one third are recruited from each of the core entry pathways [15]. Upon completion of a core training pathway, candidates are eligible for the four-year specialist training program.

Competition

The core training pathway competition varies from year to year. In 2024, there were 3.67 applicants per place for Internal Medicine Training (IMT), 3.69 for General Practice and 7.57 for ACCS Emergency Medicine [16]. In the same year, there were 8.56 applicants per available Sports and Exercise Medicine ST3 post. The ratio of applicants has risen rapidly from 2020 at 2.91 applicants per post to 5.83 in 2021 and 9.00 in 2022; however, this has been a result of fewer available posts [17]. In 2020, 11 posts were available compared to just five in 2021 and six in 2022, hence a spike in the competition ratio. With growing interest in the specialty, competition ratios remain high despite nine posts available in 2024 reflecting an increased volume of applicants.

Due to the highly competitive nature of SEM, applicants need to ensure a comprehensive portfolio to increase their likelihood of success. The selection process and the panels favour applicants who have shown commitment and interest in the specialty. Applicants are shortlisted based on a national selection specification where scoring incorporates postgraduate qualifications, clinical experience, academic skills and involvement in research, in addition to teaching experience and leadership [18]. High-scoring applicants are then invited to interview. The interview process consists of three structured stations: suitability and commitment to the specialty, a clinical scenario and an ethical scenario. Each station is scored out of 5 by two assessors, giving a raw interview score out of 40, which, once weighted and combined with the portfolio score (scaled to a maximum of 20), produces a final ranking score out of 100 [18].

Training Requirements

SEM trainees embark on a four-year higher specialty training pathway designed to provide a comprehensive exposure to a wide spectrum of conditions and clinical settings [15]. It is structured to address individual learning needs and builds upon prior experience gained during core training. Key components include substantial musculoskeletal experience, general practice, public health and emergency medicine. Trainees must also gain experience in specialist clinics (e.g., pain and orthopaedics), rehabilitation, sport-specific environments and relevant medical specialties such as cardiology and rheumatology. These diverse placements ensure trainees develop the broad and multidisciplinary capabilities needed in SEM.

To progress, trainees must demonstrate capability in all curriculum domains via workplace-based assessments, ARCP and an up-to-date portfolio [15]. Medical graduate portfolios are records of trainees' learning and clinical experience, serving as evidence of progression. They include the trainee's clinical attachments, reflective practice, academic work, formal assessments and extra-curricular involvement. By ST5, the trainee must have completed the faculty membership exam in SEM. In addition, trainees must complete relevant acute life support courses (e.g., ALS, pitch side trauma care), fulfil research and audit requirements, engage in teaching and leadership activities, and maintain GMC revalidation [15]. Upon satisfactory completion of training and all assessments, trainees are awarded a Certificate of Completion of Training (CCT) in SEM and are eligible for inclusion on the specialist register.

United States of America

The role of Sports and Exercise Medicine is becoming increasingly crucial in the US, with poor promotion of physical activity nationally and a limited emphasis on exercise medicine in graduate medical programs [19]. With MSK complaints accounting for nearly one in five primary and emergency department visits in the United States, there is a growing demand for clinicians specifically trained in non-surgical musculoskeletal assessment, injury prevention and rehabilitation [20,21]. The United States also has one of the most developed ecosystems of organised sport globally, with collegiate athletics alone accounting for over 500,000 student-athletes and serving as a direct pipeline to elite-level sport [22]. In this context, SEM physicians play a role in both tackling the chronic disease burden as well as optimisation of athletic performance in competitive sport [23,24]. This reflects the growing relevance in both general and elite medical practice.

Training Pathway

Sports medicine is a subspecialty in the United States [25]. Sports physicians stem from the following approved core primary specialties: family medicine, internal medicine, emergency medicine, paediatric medicine and physical medicine and rehabilitation (PMR) [26]. Additionally, there are surgical sports medicine fellowships following the orthopaedic route, not covered in the scope of this paper. In the US, students are required to complete a bachelor's degree before admission to a postgraduate medical program. Following graduation from medical school, prospective applicants must match into a residency program in the aforementioned core specialties via the National Resident Matching Program (NRMP). After completion of residency, physicians from these primary specialties apply for additional training in Sports Medicine through accredited fellowship (subspecialty) programs [27].

Fellowships are available across the country and the majority of posts are applicable via a centralised electronic approach. Select programs require direct application and criteria. All posts are in conjunction with the American Medical Society for Sports Medicine (AMSSM) and are accredited by the Accreditation Council for Graduate Medical Education (ACGME) [27]. Applicants can apply in their final year of residency or after having successfully completed the residency program. Fellowships are typically recognised as a subspecialty of the applicant’s primary board and lead to a Certificate of Added Qualification (CAQ) in Sports Medicine upon completion of further examination and successful completion of training.

Similarly, fellowship posts are matched via the NRMP. Selection criteria are specific to each fellowship post and are determined by the fellowship provider, but common criteria taken into consideration include the United States Medical Licensing Exam (USMLE) scores, curriculum vitae (CV), personal statement, letters of recommendation, experience in sports medicine, research involvement and extracurricular involvement [28,29]. There is no standardised nationwide scoring system like the UK’s selection criteria for training posts.

Competition

Sport Medicine historically attracts more applicants than available places demonstrating the competitive nature of fellowship posts. According to 2025 NRMP match data, there were 392 Sport Medicine fellowship positions with a fill rate of 99% [30]. Of these programs, 302 were sponsored and filled by the Family Medicine pathway. The total number of applicants has steadily increased over recent years, reflecting growing interest in the specialty, while the number of available positions has remained relatively stable [31]. Whilst the overall competitiveness for sports medicine as a subspecialty remains high, well-established academic programs offering event coverage with professional sports teams tend to be most sought after.

Whilst any applicant is eligible to apply provided board certification requirements are met, there are some ways an applicant can evidence an increased commitment to the specialty. An elective in sports medicine, physician experience with local high school teams, medical coverage in mass participation event, presentations at accredited sports medicine conferences, publications in the field of sports medicine and attendance at sports medicine conferences can all demonstrate increased commitment [32].

Training Requirements

The majority of fellowships are 12-month clinical programs designed to provide broad exposure to non-surgical musculoskeletal (MSK) care, sport-related injuries, exercise prescription, and team and event coverage [33]. Fellows can often receive training in musculoskeletal ultrasound, physical rehabilitation, orthopaedic medicine, cardiopulmonary physiology, concussion management and public health aspects of physical activity promotion. Most programs include rotations in MSK radiology, adult and paediatric orthopaedics, rheumatology, rehabilitation medicine, cardiology and emergency medicine. Fellows are expected to develop competencies in both acute injury management and chronic overuse conditions. Programs often integrate sideline and event coverage providing experience with athletes at various levels.

Fellows must complete all ACGME program requirements and pass their subspecialty board examinations, which are administered by the candidate’s primary specialty board, to obtain a CAQ in Sport Medicine [25]. Fellowship completion also requires engagement in research or academic activity, ongoing assessment through workplace-based evaluations, and participation in teaching and leadership activities. Certification must be maintained through continuing revalidation in line with the primary board’s requirements [25].

Canada

Training Pathway

Similar to the US system, Canadian students are generally required to complete an undergraduate degree prior to medical school admission. The Canadian system also provides training opportunities for SEM in the form of a subspecialty. Medical graduates who are pursuing SEM most commonly undertake the family medicine route, completing a two-year residency program [34]. A 12-month-long enhanced skill residency in SEM is offered by the College of Family Physicians of Canada (CFPC) [35]. Upon completion of the residency, physicians are awarded a Certificate of Added Competence (CAC) and become eligible to sit a diploma in SEM provided by the Canadian Academy of Sport and Exercise Medicine (CASEM) [34].

Candidates can also pursue SEM through specialist pathways such as Internal Medicine, Emergency Medicine, Physical Medicine and Rehabilitation (PM&R), Paediatric Medicine and Orthopaedic Surgery. These physicians may pursue SEM as an Area of Focused Competence (AFC) through additional training in SEM integrated into their residency (with program director approval) or via a separate Royal College-accredited extra fellowship year upon residency completion [34]. AFC certification in SEM is provided by the Royal College of Physicians and Surgeons of Canada.

Competition

Those in their second year of family medicine residency are eligible to apply for the enhanced skills residency. Programs typically shortlist applicants based on academic performance in core family medicine training, commitment to SEM, references and interview performance. Enhanced skill residencies are offered by 14 universities, each typically providing only 1-2 positions [36]. While official competition data is not publicly available, the limited number of placements reflects the competitive nature of these posts. Similarly, there is no published data on AFC training, and routes outside of family medicine are considered non-traditional and significantly less common.

Training Requirements

The Enhanced Skills Residency in SEM in Canada is tailored to the CFPC’s Priority Topics and Key Features for SEM [37]. Residents are expected to demonstrate proficiency across a variety of domains, including musculoskeletal assessment and management, concussion care, exercise prescription, radiological interpretation, procedural skills (e.g., joint injections and ultrasound-guided interventions), and return-to-play decision making. Competencies are not limited to just clinical categories but span communication, collaboration with allied health professionals and leadership in multidisciplinary team environments. Resident assessment is competency-based workplace-based assessments (WBAs), e.g., direct observations, field notes, supervisor evaluations and colleague feedback [37]. Upon program completion, candidates are eligible to receive the CFPC Certificate of Added Competence (CCFP-SEM) and may sit the OSCE-styled CASEM diploma in SEM.

AFC training delivered by the Royal College of Physicians and Surgeons of Canada is structured based on the college’s Competency Training Requirements (CTR) with a similar focus to the SEM enhanced skill residencies [34]. A Royal College Diploma in SEM (Dip SEM RCPSC) is issued upon completion. Graduates may also sit the CASEM Diploma in SEM if their training meets eligibility requirements [36].

Australia and New Zealand

Training Pathway

There is a single direct route into SEM training in Australia and New Zealand, and unlike the UK, there are no core specialty feeder pathways. Medical graduates must first complete a recognised undergraduate or postgraduate, followed by a mandatory one-year internship (PGY1) to gain general registration with the Australian Health Practitioner Regulation Agency (AHPRA) or the Medical Council of New Zealand (MCNZ), respectively [38,39]. The Australasian College of Sport and Exercise Physicians (ACSEP) oversees Sports and Exercise Medicine training in both Australia and New Zealand, along with recruitment into the specialist training [40]. Candidates must have a minimum of three years full-time postgraduate clinical ensuring a broad-based clinical foundation, working in hospital or general practice environments, to be eligible for the ACSEP training program. Prospective SEM trainees must also pass the ACSEP entrance examination for eligibility to apply for the training program. The entrance test is a written test that assesses the candidate’s medical knowledge relevant to sport and exercise medicine [40].

Competition

If the candidate fulfils the eligibility criteria for the application, they must then submit a formal application to the program. This involves submission of a comprehensive CV, referee reports and evidence of clinical and academic experience [41]. The CV is assessed per domain, each with varying weighting. Work history and ACSEP engagement each contribute the highest weightings at 25.5%. Work history includes SEM-related clinical placements, while ACSEP engagement includes attendance at conferences, completion of SEM-specific education modules, awards and personal participation in SEM. Sports and exercise work history pertains to experience in SEM specific environments and holds a further 21.3%. Academic achievements, including postgraduate qualifications, account for 8.5%, and leadership and community engagement also contribute 8.5%. Research involvement contributes 6.4% with first author publications particularly valued and presentations contribute a further 4.3% [41].

Shortlisted applicants are invited to a Multiple Mini Interview (MMI) format, with several structured interview stations to assess suitability for SEM. Stations range from 6-10 minutes and possible domains include communication, collaboration, management, quality and safety, health advocacy, research, teaching and learning, professionalism and cultural awareness [40].

ACSEP training post selection statistics are not commonly published. The vigorous selection process reflects that posts are limited with high competition. According to the 2024 ACSEP annual report, there was a 65% increase in applications in the 2024 application cycle, with 43 applications for 18 registrar posts [42].

Training Requirements

The ACSEP Specialist Training Program can be completed in three years and seven months on a full-time program [43]. The program prepares registrars to deliver comprehensive patient-centred care across the full spectrum of sport and exercise medicine with rotations in musculoskeletal medicine, rehabilitation, public health and general medicine. Furthermore, exposure to sports team coverage, exercise prescription and event medicine is integral to the program. By completion, trainees are expected to manage acute and chronic injuries, prescribe exercise for prevention and disease management, and address the needs of diverse populations including children, older adults and para-athletes [43].

Registrars are supervised by accredited ACSEP Fellows and must complete structured workplace-based assessments, clinical logs and academic requirements [40]. Trainees must also undertake and pass the ACSEP Fellowship Examination in addition to maintaining continuing professional development via research projects, completion of courses and involvement in teaching activities. Progression is monitored through regular reviews and submission of portfolio evidence. Upon satisfactory completion of all training requirements and successful passing of the Fellowship Examination, registrars are awarded Fellowship of the Australasian College of Sport and Exercise Physicians (FACSEP) with entry onto the specialist registrar.

Training pathways in Sports and Exercise medicine differ markedly across the globe. The UK, Australia and New Zealand recognise SEM as a stand-alone specialty, delivered through formalised national training programs. In contrast, the US and Canada integrate SEM as a subspecialty following completion of a core residency, delivered through shorter fellowship programs.

Training duration varies by country and pathway, as depicted in Table 1. The UK adopts the longest route to full qualification, requiring a minimum of 8-9 years depending on the core training route [15]. This includes both a core specialty and a high specialty training program. This is followed by Australasia, adopting a 7-year training route which does not require candidates to have completed a core specialty but does mandate three years of postgraduate experience prior to entry into the ACSEP program [40]. Longer training pathways delay independent practice and subsequent consultant-level earning potential. Canada offers the shortest training route for those entering SEM via a two-year family medicine residency followed by a one-year enhanced skills program year, totalling three postgraduate years [34]. Please refer to Table 1 and Table 2 for the summary and key features of each training pathway per country.

**Table 1:** Outline of the Sports and Exercise Medicine Training Pathways After Medical Graduation Across Five English-Speaking Countries * Undergraduate degrees are required in the US and Canada prior to medical school admission ** Enhanced Skills Residency Program GP: General Practitioner; ACCM: Acute Care Common Stem; IMT: Internal Medicine Training; PMR: Physical Medicine and Rehabilitation; ACSEP: Australasian College of Sport and Exercise Physicians; FY: Foundation Year; PGY: Postgraduate Year; ACCS: Acute Care Common Stem; IMT: Internal Medicine Training; CAQ: Certificate of Added Qualification; CAC: Certificate of Added Competence; SEM: Sports and Exercise Medicine.

Total Number of Training Years	Country	UK			USA*					Canada*		Australia + New Zealand	
	Training Route	GP	ACCS (EM/ IM)	IMT	Family Medicine	Internal Medicine	Emergency Medicine	PMR	Paediatrics	Family Medicine	Royal College Route (IM / PMR / Paediatrics/ EM)	ACSEP	
1	Postgraduate Years	FY1			PGY1	PGY1	PGY1	PGY1	PGY1	PGY1	PGY1	PGY1 (internship)	
2		FY2			PGY1	PGY1	PGY1	PGY1	PGY1	PGY2	PGY2	PGY2	
3		GPST1	ACCS1	IMT1	PGY3	PGY3	PGY3	PGY3	PGY3	ESRP**	PGY3	PGY3	
4		GPST2	ACCS2	IMT2	Fellowship	Fellowship	(PGY4)	PGY4	PGY4	-	PGY4	ACSEP 1	
5		GPST3	ACCS3	ST3	-	-	Fellowship	Fellowship	Fellowship		(PGY5)	ACSEP 2	
6		ST3		ST4			-	-	-		RCPSC-accredited SEM Subspecialty Residency	ACSEP 3	
7		ST4		ST5								ACSEP 4	
8		ST5		ST6							-	-	
9		ST6		-									
-	Qualification Title	Certificate of Completion of Training (CCT)			Board Certification + CAQ in Sports med					CCFP + CAC in SEM	FRCPC + SEM Subspecialty (RCPSC)	Fellowship of ACSEP	

**Table 2:** A Comparison of the Key Features of Sports and Exercise Medicine Training Programs Across Five English-Speaking Countries *Number of SEM Fellows via the CFPC Pathway Estimated number of practising SEM consultants [37, 44-46] GMC: General Medical Council; FSEM: Faculty of Sports and Exercise Medicine; JRCPTB: Joint Royal Colleges of Physicians Training Board; AMSSM: American Medical Society for Sports Medicine; ABMS: American Board of Medical Specialties; CFPC: College of Family Physicians of Canada; ACSEP: Australasian College of Sport and Exercise Physicians; CAQ: Certificate of Added Qualification; CAC: Certificate of Added Competence; SEM: Sports and Exercise Medicine; FACSEP: Fellowship of the Australasian College of Sport and Exercise Physicians; JRCPTB: Joint Royal Colleges of Physicians Training Board; IMT: Internal Medicine Training; GP: General Practitioner; ACCS: Acute Care Common Stem; PM&R: Physical Medicine and Rehabilitation; FSEM: Faculty of Sports and Exercise Medicine; CASEM: Canadian Academy of Sport and Exercise Medicine.

A comparison of key features in each country					
-	UK	USA	Canada	Australia	New Zealand
Qualification body	GMC / FSEM/ JRCPTB	ABMS / AMSSM	CFPC / RCPSC	ACSEP	ACSEP
Qualification name	Certificate of Completion of Training (CCT)	Board Certification + CAQ in Sports Medicine	CCFP + CAC in SEM	FACSEP	FACSEP
			FRCPC + SEM Subspecialty (RCPSC)		
Organisation developing the curriculum	JRCPTB	AMSSM	CFPC / RCPSC	ACSEP	ACSEP
Estimated number of practicing SEM consultants	200	3800	352*	177	43
Pre-Med Undergraduate Degree required	N	Y	Y	N	N
Pre-Med duration	-	4	3-4	-	-
Medical school duration (direct entry)	5-6	4	4	5-6	5-6
Number of post graduate years of training (minimum)	8	4-5	3(FM) 6-7 (specialist)	7	7
Core specialty required	Y	Y	Y	N	N
Core specialty routes (if applicable)	GP/ IMT/ ACCS (EM)	Family Med, IM, PM&R, EM, Paeds	Family Med OR IM/ Paeds/ PM&R	N/A	N/A
Training program or subspecialty	Training Program	Subspecialty	Subspecialty	Training program	Training program
Years of SEM training program (if applicable)	4	N/A	N/A	4	4
Number of training positions for 2025 entry	9	-	-	18	
Number of fellowship positions	-	392	-	-	-
Number of universities offering fellowships	-	-	14	-	-
Licensing exams	MFSEM	Fellowship exam	Local fellowship exam / RCPSC SEM Subspecialty Exam	ACSEP	ACSEP
Additional exams	FSEM Diploma Examinations	-	CASEM diploma	-	-

The prior residency model adopted by the US and Canada ensures clinical maturity and a broad clinical foundation to build upon prior to SEM-specific training. Fellowships are typically one year focusing predominantly on musculoskeletal medicine with less emphasis on exercise medicine and public health involvement [25]. These programs are not standardised nationally introducing variability to course structure, content and quality. The short nature of fellowships and minimal previous SEM training does result in limited clinical exposure. Some programs rotate trainee placements very frequently to cover the required learning requirements. Fellowships tend to be procedure-heavy with focused training on ultrasound.

Full completion of prior residency produces SEM physicians with a well-developed skill set in their base specialty. Whether that is the acute care and decision-making stemming from emergency medicine, the continuity and management of chronic disease in family medicine or the interventional rehabilitation of PM&R, trainees inherit a specialised foundation. On the other hand, shorter SEM programs reduce depth throughout the program with a reduced public health focus. The US fellowships, in particular, exhibit a severe lack of emphasis on exercise medicine, with 63% of fellows reporting the absence of structured teaching in exercise prescription throughout their programs [19]. Conversely, stand-alone SEM models allow for sustained focus on all SEM domains but lack the breadth of pre-existing specialist experience. Furthermore, the stand-alone specialty model provides a clear SEM identity on specialist registers, whereas fellowship models offer dual identity with greater fallback options in the base specialty, in case of reduced job market opportunities.

With established collegiate and professional sports systems in a wide variety of sports, US fellowships generally offer excellent team medicine exposure, including medical coverage participation for mass events. Canada and Australasian programs also integrate substantial team medicine involvement, but this is more dependent on the host university’s affiliation and the regional sports ecosystem.

The structured training programs in the UK and Australasia ensure continuous SEM exposure over four years, providing greater depth across all domains and broadening programs beyond a musculoskeletal medicine focus [15,36]. This breadth supports SEM roles beyond sports, incorporating public health involvement, exercise physiology and rehabilitation in a variety of clinical settings. Training programs adopt a multidisciplinary team focus with integrated placements in closely related specialties relevant to sports and exercise medical practice [47]. Furthermore, a nationally approved and standardised curriculum reduces variability amongst trainees with clear objectives and assessment milestones [15,36]. Protected study time is often built in for research and academic activity, enabling trainees to contribute to the evolving evidence base of the specialty.

Program availability also varies significantly. The UK has extremely limited posts and high competition with successful applicants often holding postgraduate qualifications, team-coverage experience, and research and teaching portfolios [18]. Canada, similarly, exhibits limited fellowship spots in high demand, with only 14 institutions offering places [36]. Australia and New Zealand also experience oversubscription to the training program, but with slightly better odds. The latest ACSEP cycle saw 43 applicants with 18 recruited as new registrars [42]. In comparison, the UK received 77 applications for nine available posts [16]. The US offers the largest number of fellowship programs, adopting a centralised application process [30]. However, given programs exhibit variability, competition remains strongest for the most reputable and geographically desirable programs.

## Conclusions

Postgraduate training in Sports and Exercise Medicine varies significantly across the UK, US, Canada, Australia and New Zealand. Variability reflects differences in the healthcare system, public health demands, sporting cultures, role of the SEM consultant and recognition of the specialty. Stand-alone specialty models deliver structured and comprehensive exposure to SEM over multiple years with multi-specialty exposure and experience across multiple clinical settings. National programs adhere to organised domains of learning and clear criteria for meeting learning requirements. These programs tend to be longer in duration with fierce entry competition, but prepare trainees for leadership roles in multidisciplinary teams and integrate exercise medicine into healthcare delivery. Fellowship models provide shorter and often procedurally rich routes with strong team coverage opportunities but lack clear scope and depth, often focusing on musculoskeletal medicine. Curriculum variability and the limited emphasis on exercise medicine may be drawbacks for those seeking a more comprehensive role in chronic disease management. Each approach carries strengths and drawbacks in relation to training length, application requirements, curriculum content and competition for entry. By identifying these differences, this review provides a framework to inform potential refinements to existing training pathways and supports aspiring SEM trainees to select a route aligned to their progressional goals and the competencies required to succeed in both the application process and subsequent training. Future reforms should aim to standardise the role of the Sport Physician and establish a unified competency framework to guide training programs. Given the variations in training pathways and core specialty foundations, achieving global recognition of qualifications remains challenging. International collaboration and universal competency frameworks can enhance global mobility amongst SEM specialists whilst aligning training with the evolving demands of modern healthcare systems.
